# Malignant Melanoma and Its Stromal Nonimmune Microecosystem

**DOI:** 10.1155/2012/584219

**Published:** 2012-06-28

**Authors:** Gérald E. Piérard, Claudine Piérard-Franchimont, Philippe Delvenne

**Affiliations:** Department of Dermatopathology, Liège University Hospital, 4000 Liège, Belgium

## Abstract

In recent years, rapid advances were reached in the understanding of a series of biologic signals influencing cutaneous malignant melanoma (CMM) cells. CMM is in close contact with a peculiar dermal extracellular matrix (ECM). Stromal cells store and release various structural ECM components. The impact on CMM growth and progression is mediated through strong and long-lasting effects of ECM products. This paper summarizes some peculiar aspects of the peri-CMM stroma showing intracytoplasmic loads in Factor XIIIa, CD34, versican, and **α** (IV) collagen chains. The restricted peri-CMM skin territory exhibiting such changes corresponds to the area showing neoangiogenesis and extravascular unicellular metastatic spread. The latter inconspicuous migratory CMM cells possibly correspond to CMM stem cells or to CMM cells with aberrant HOX gene expression. Their presence is associated with an increased risk for metastases in the regional sentinel lymph nodes. In conclusion, the CMM-stroma connection appears crucial to the growth regulation, invasiveness and initial metastatic spread of CMM cells. Although much remains to be learned in this field, the active intervention of the peri-CMM stroma is likely involved in the inconspicuous early metastatic migration of CMM cells.

## 1. Introduction

Human cutaneous malignant melanoma (CMM) basically corresponds to an uncontrolled overgrowth of neoplastic melanocytes. However, the extracellular matrix (ECM) and the connective tissue cells adjacent to the neoplasm probably represent more than passive bystanders in CMM. The biology and prognostic significance of some tumoral components are increasingly established [1, 2], but the relationship between CMM cells and the nonimmune aspects of the peritumoral stroma remains poorly understood. The CMM-stroma cross-talk is regulated by soluble factors and by direct cell-cell contact [3]. It is a commonplace to suggest that CMMs with little fibroblasts are quite aggressive, but this assumption is not firmly rooted in evidence-based observations. The influence on metastatic spread is unsettled.

The aim of this paper is to summarize the evidence on the changes in the nature of stromal cells and their products in the peri-CMM stroma during tumor invasion and metastatic dissemination.

## 2. Pathobiology of Regular and ****Desmoplastic Melanomas

At a key point in tumor progression, CMM starts to express a high metastatic potential before causing mortality. In order to form distant metastases, malignant cells have to acquire specific functions and properties in an orderly sequence [4]. In particular, some interactions exist between CMM cells and various biologic systems including environmental factors, immune cells, vascularity, contiguous stromal cells, and molecular components of the dermal ECM [5–7]. Some aspects of the CMM-stroma interactions including the release of growth factors and matrix metalloproteinases (MMP) are likely associated with the disease progression and prognosis [7–11].

Cell functions required in the CMM metastatic cascade are not limited to intrinsic properties of the neoplastic cells. Many properties are regulated or influenced by the close microenvironment [12, 13]. In addition, neoplastic cells are able to induce changes in the surrounding tissues [14, 15]. Thus, CMM cells and their surrounding stroma jointly form a specific and complex microecosystem [10, 16]. Cross-talk between CMM and stromal cells is likely mediated through a combination of direct heterotypic cell-cell contacts, adhesion molecules, signaling factors, as well as other secreted molecules including growth factors, cytokines, chemokines, ECM proteins, MMPs, proteinase inhibitors, and lipids [3, 17]. Conceptually, the CMM microecosystem is crucial for the maintenance of both the cell functions and tissue integrity suggesting that any CMM-induced change in the stroma contributes to the neoplastic progression and invasiveness [18]. Any alteration in the CMM stroma is likely related to various imbalances in the cytokine profile, resulting from the oncogenic changes in the neoplastic cells. In particular, experimental animal models demonstrated that cancer invasion was stimulated by the wound-healing stroma [19].

Both stromal cells and ECM components located beneath primary CMM are therefore involved in both the neoplastic invasive process and the early dissemination of micrometastases associated or not with neoangiogenesis [2, 20–23]. These characteristics are expected to be related to phenotypic changes in the stromal cells in the CMM vicinity [10].

The three-dimensional relationship between neoplastic cells and stroma varies among tumors. In benign tumors, a clear cut separation exists between neoplastic cells and the connective tissue. By contrast, especially in malignancies, areas of tumoral cells and stroma are merging, since the former ones often invade the latter structures.

Desmoplastic melanoma is a rare and specific cytomorphologic variant in the CMM spectrum [24]. This neoplasm exhibits a clinical behavior distinct from regular CMM, including frequent local recurrences. The neoplastic melanocytes commonly exhibit unusual poorly differentiated phenotypic characteristics. Microscopic features of desmoplastic melanoma are distinct from regular CMM. They consist of invasive neoplastic spindle-shaped melanocytes encased in a striking desmoplastic stroma. Fibroblast-like cells are intimately admixed with the neoplastic melanocytes. They exhibit either a single-cell dispersal pattern within the sclerotic dermis or are disposed in long fascicles. Desmoplasia is commonly related to a variety of cell lines including numerous Factor XIIIa+ dermal dendrocytes (DDs) and rare *α*-actin+ myofibroblasts (MFs). In addition, CD45+ lymphocytes and Mac 387+ monocytes/macrophages are clustered inside desmoplastic melanoma.

## 3. Tumor Cell Involvement in Micrometastases

The primary CMM thickness impacts the prognosis of the neoplasia. Primary CMMs less than 1 mm thick are associated with high cure rate and unaffected survival. This situation sharply contrasts with the poor prognosis of thicker CMM. The apparent breakpoint beyond about 1 mm thickness is a dramatic aspect for the disease outcome. One possible reason appears to be linked to the CMM vascularization patterns [25–28]. Such microanatomic feature is persuasive, but it is by no means the single one [25].

The concept of dormant CMM metastases [29] was offered to explain (a) why some CMMs develop metastases after disease-free intervals as long as 10–40 years following excision of the primary CMM, (b) the therapeutic failure of wide surgical resection margins, and (c) the vain hope placed in elective lymph node dissection to influence prognosis. Once CMM metastases have developed, the subsequent prognosis is fairly predictable, irrespective of the preceding disease-free interval. These observations suggest that microscopic CMM foci likely remain quiescent in their own microenvironment for variable periods of time before some event(s) trigger(s) the development of detectable/progressive metastases.

The metastatic potential is expressed by variant subpopulations of metastatically competent CMM cells present in the primary neoplasms [2]. These subpopulations typically exhibit over time a growth advantage at the primary site leading to a dominant proliferating population [2]. At this stage, CMMs express overt malignancy and clonal dominance of metastatically competent cells. Most thin primary CMMs only contain few, if any, metastatically competent cells, whereas thicker CMMs contain increased proportions of such cells. The stromal microecosystem is in part involved in such a shift in the biologic profile [30].

### 3.1. Melanoma Stem/Progenitor Cells

The presence of CMM stem cells is in part involved in the process of CMM micrometastases and in their relationship with the peritumoral stroma [20, 31–36]. CMM stem cells are capable of indefinite self-renewal, a function linked to CMM growth [2, 34, 37]. Although conventional anticancer treatments aim at eradicating malignant cells, they are potentially ineffective against chemoresistant cancer stem cells, which are ultimately responsible for neoplastic recurrence, progression, and metastasis [2, 20, 31].

CMM typically exhibits cell heterogeneity, undifferentiated molecular signatures, and increased tumorigenicity restricted to specific CMM subsets with embryonic-like differentiation plasticity. These features strongly suggest the involvement of CMM stem cells in the initiation and spreading of this malignancy [31–33, 35, 38–41]. Both the structure and biologic specificity of the ECM are potentially involved in the invasiveness and propagation of CMM stem cells.

### 3.2. The Metastatic Paths

The generation of CMM metastases at distant sites relies on adequate genotypic and phenotypic characteristics acquired in an orderly sequence referred to as the CMM metastatic cascade. Such a process encompasses boosted CMM cell proliferation, detachment from the primary neoplasm, interaction with and invasion into the peritumoral stroma, penetration into blood and lymphatic vessels, survival in the circulation, adhesion to a vessel wall at the final metastatic site, extravasation, survival in the new ECM, and proliferation to form overt metastasis [2, 5]. Thus, basically, the specific functions involved in the metastatic cascade combine intrinsic characteristics of some CMM cells and the regulatory influences from the microenvironment. Indeed, CMM cells and their surrounding stroma jointly form a microecosystem receptive to early inconspicuous metastatic spread [8].

The early CMM micrometastases are poorly discernable at the regular clinical or dermoscopic examinations. By contrast, most of them are disclosed under the microscope, particularly with the help of immunohistochemistry [2, 10, 22, 42]. They are found in four main distinct locations, namely, (a) inside lymph vessels, (b) inside blood vessels, (c) in a perivascular location just abutted to the outer aspect of the endothelial lining, and (d) dispersed inside the stroma [20, 21, 23]. The latter eventuality is frequently associated with neoangiogenesis, and a boosted neoplastic germinative pool is commonly found [20, 28].

The active migration of metastatic CMM cells in the peritumoral stroma is probably a complex process. It involves both the active migration of CMM cells and changes in the CMM cell adherence systems with ECM components. Neoplastic thigmotropism is in part involved in the migration path [43].

Interstitial unicellular or paucicellular CMM micrometastases are occasionally found and confined to the perineoplastic stroma. Their presence is statistically correlated with the risk of invasion of the sentinel lymph node [22]. Microarray studies have identified some transcriptional differences during the metastatic cascade [4]. Of note, overexpression of some homeobox (HOX) genes is typically present in CMM cells susceptible to generate metastases. This facet of CMM pathobiology conveniently revealed using immunohistochemistry shows correlation with specific changes in the peritumoral stroma as described hereafter. The role of homeobox (HOX) gene overexpression has been highlighted in the evolving process of CMM metastasis [44–47]. In addition, the MMP expression is altered. Although MMPs are typically degradative enzymes with the ability to cleave ECM proteins such as collagen and fibronectin, their dominant role is actually a signal-altering effector [48]. These nonmatrix-degrading functions of MMPs vastly expand the ways in which they contribute to various pathologies, including CMM progression.

## 4. Stroma Immunohistochemistry Abutted ****to Malignant Melanoma

At the regular histopathology examination, stromal cells look normal underneath primary CMM when partial regression is not obvious. However, their differentiation as revealed by immunohistochemistry appeared altered when compared to the surrounding skin. In particular, phenotypic changes were found in the expression of the transglutaminase Factor XIIIa, CD34, the *α* (IV) collagen chains, the *α*- and *β*-actins, as well as elafin and versican. It is possible although not yet proven that transforming growth factor-*β*1 (TGF-*β*) and platelet-derived growth factor (PDGF) play a role in changing the stromal host compartment in CMM.

### 4.1. Factor XIIIa-Enriched Stromal Cells

Factor XIII exerts a major function in the area of hemostasis. It has a hetero-tetrameric structure which consists of two globular A units and two strandlike B subunits. The A subunit is a proenzyme circulating in the blood and present intracellularly in platelets and monocyte-macrophages.

Factor XIIIa-enriched stromal cells of the skin are commonly identified as DDs type I. The intracytoplasmic subunit A is a specific transglutaminase [49]. The cells belong to the monocyte-macrophage lineages preferentially abutted to the superficial microvasculature [50–52]. Increased numbers of Factor XIIIa+ DD are often found in the vicinity of most invasive cutaneous neoplasms. They are particularly numerous, abutted to and infiltrating most thin CMM [30]. By contrast, they are fewer or even absent in thick primary CMM and their metastases [52–55]. Circumstantial evidence correlated the density of Factor XIIIa+ DD and a low proliferative rate of CMM cells [30]. Thus, Factor XIIIa+ DD are probably not passive bystanders in CMM [29, 48, 49]. Their function possibly differs based on whether they are located in the stroma or inside the neoplasm. Intratumoral DD appeared associated with a growth-restricting role. In contrast, stromal DDs contribute to the invasiveness and metastatic spread of CMM cells. This role in CMM invasiveness is putative but it unambiguously opens new lines of inquiry about the biologic functions of Factor XIIIa.

### 4.2. CD34-Positive Stromal Cells

CD34+ type II DDs are constitutive cells of the dermal stroma. Invasive CMMs are frequently associated with a decrease in these cells [56].

### 4.3. Collagen-IV-Enriched Stromal Cells

In malignant neoplasms, basement membranes (BMs) are composite structures synthesized by tumoral cells, stromal cells, by one of these two cell types, but depending on interactions between them, or are a mixture from both origins. These tumor-associated BMs are often abnormal in their composition and ultrastructure [57, 58]. BM material seems rather accompanying malignant cells rather than preventing invasion as a physical barrier. Nevertheless, active interactions between neoplastic cells and stroma, in particular the ECM, play a key role in the neoplastic progression leading to invasion and metastasis [59]. Several BM components were identified around CMM cells including collagen IV [57, 60–62]. In the skin, collagen IV represents an assembly of *α*1 (IV) and *α*5 (IV) collagen chains. In CMM, some neoplastic cells and stromal cells exhibit intracytoplasmic immunolabeling for *α*1 (IV) chains [62]. The pattern is heterogeneous, and BM components including type IV collagen gradually disappear during the dermal ingrowth of CMM cells. Of note, a minority of CMM without any identifiable micrometastasis and a majority of CMMs with cutaneous micrometastasis show discrete cytoplasmic positivity for the *α*5 (IV) collagen chain [60].

The distribution of *α*1 (IV) collagen chain in CMM highlights the heterogeneity in both cell differentiation and stroma-CMM interactions. Thus, CMM cells appear to have their own individual potential to be wrapped by a BM and to interact with the stroma. This biologic aspect is likely related to the neoplastic progression and it influences the inconspicuous metastatic potential.

### 4.4. Versican-Enriched Stromal Cells

Versican is a large proteoglycan normally present inside stromal cells of the skin. The molecule belongs to the chondroitin sulfate family of the hyalectan group, named from its ability to bind to hyaluronan [63]. In mammals, versican appears as four possible spliced isoforms named V0 to V3. Little is known about the differential regulation of the isoforms and about their respective roles in the ECM either normal or peritumoral. The versican production is deregulated in several human cancers [64]. As it is largely expressed in fast-growing neoplastic cells, it has been suggested to play a direct role in cell proliferation and other cell functions [63]. It appeared to be particularly abundant in the stromal cell population underneath CMM [64–71]. The overexpression was sharply circumscribed to a cup-shape structure cuffing the bottom of CMM. In addition, some nests of CMM cells were strongly labeled with the antiversican antibody. Such finding contrasts with another study having reported the absence of versican immunoreactivity in neoplastic melanocytes [67]. Versican expression did not correlate with Breslow tumor thickness and Clark's level [67].

### 4.5. Protomyofibroblasts and Myofibroblasts

The peritumoral stroma of some CMMs contains both MF and their precursors, the protomyofibroblasts. Any MF resembles a peculiar fibroblast-like cell, exhibiting features of a smooth muscle (SM) cell, including microfilament bundles, with interspersed dense bodies, and intercellular gap junctions. MFs are present in most fibrotic conditions. The MF structure is characterized by stress fibres formed by contractile proteins, particularly *α*-SM actin (*α*-SMA). MFs originate from protomyofibroblasts whose stress fibers contain *β*- and *γ*-actins. They possibly evolve to regular MFs enriched in *α*-SMA [72].

The switch from the protomyofibroblast to mature MF probably results from the production and release of transforming growth factor-*β*1 (TGF-*β*1) by inflammatory and fibroblast-like cells. The TGF-*β*1 action is mediated by the cellular fibronectin splice variant ED-A [72]. Thus, MF differentiation is regulated by both specific cell components and some ECM molecules. Endothelin-1, thrombin, and mechanobiology promote MF induction [73, 74].

Contrasting with melanocytic nevi and desmoplastic melanomas, the stroma of most regular CMM appears to be devoid in MF exhibiting *α*-SMA immunoreactivity [56, 75]. *β*-SMA+ protomyofibroblasts are rare as well.

### 4.6. Elafin-, Versican-, and Lysozyme-Loaded ECM

Elastic fibres are coated by distinct molecules following chronic ultraviolet light exposures [76–78]. The serine antileukoprotease elafin, and versican and lysozyme as well bind to elastin preventing elastolytic degradation by elastases on sun-exposed areas exhibiting solar elastosis [77–79]. Elastase inhibition possibly decreases the adhesion of cancer cells to endothelial cells [80].

In addition, elafin was reported to elicit p53-dependent apoptosis in cultured CMM cells transfected by a plasmid producing elafin under doxycycline boosting [81]. Contrasting with such in vitro experiments, immunohistochemistry did not disclose intratumoral cell presence of elafin in human CMM. Rather, keratinocytes covering CMM overexpressed elafin in their cytoplasm. Of note, Western blot and reverse transcription analyses indicated transcriptional elafin repression in CMM cells [81].

## 5. Tumors: Wounds That Do Not Heal?

About 25 years ago, the comparison of tumors and wounds led to an intriguing concept considering the resemblance between cancers and wounds [82]. The comparison was based on molecular and biologic features occurring in wound healing on the one hand and in neoplasms on the other hand. If most observations indicate that many neoplastic cells induce stroma formation, the reverse question explored the possible induction of tumor cell development by a scar. The involvement of fibroblasts in CMM stromagenesis occurs through different stages involving their recruitment, activation, and conversion to MF, or differentiation to fibrocytes. Such involvement is topographically linked to different areas inside and around CMM, and stromal activation, as seen in tumor ulceration and immunologically-driven regression of CMM stimulating neoplastic progression [3].

## 6. Discussion

In general, the peritumoral stroma creates a kind of scaffolding for the neoplasm. In particular, a singular ECM structure is abutted to the deep part of primary CMM. Stromal cells exhibit particular phenotypic aspects suggesting altered functionality. The involved territory seems conductive to a micrometastatic spread. Some of these metastatic cells survive singly and are possibly in their way of migration to other organs. They possibly correspond to CMM stem cells or to mature CMM cells with HOX gene dysfunctions.

The intimate relationship between neoplastic cells and their stroma is a fascinating topic orienting the translational research in therapy. Beyond the importance of CMM vascularity for the tumoral growth, invasiveness, and metastatic spread [20–26, 83, 84], a series of other roles are ascribed to the tumoral stroma. Such a structure is involved in a constant remodelling following degradation and repair of the ECM. In addition, some environmental influences including ultraviolet light exposures are involved in the CMM initiation. In addition, they support the neoplastic progression through the intervention of diverse autocrine and paracrine factors [85]. Of note, immunohistochemistry highlights the direct implication of CMM cells in the synthesis and/or storage of some ECM molecular components.

The immunohistochemistry characterization of CMM cells is informative [8, 34, 86–91], but it should probably be extended to its peritumoral stroma including the microvasculature [20, 25, 26, 83], the stromal cells, and ECM components as well. A comprehensive mapping of CMM genotypic and phenotypic characteristics identifies relevant targeted therapies [89, 91–99]. Inflammatory cells and immunocytes represent another class of host cells that are regulated by cytokine balances. They perform countercurrent invasion, from the circulation into the neoplasm, and provide roads for CMM cell invasion. The global host involvement in the cancer microecosystem basically relies on the microvasculature, the stromal cells and the specific immune reactions [10, 20, 100]. Angiogenesis is a host-mediated response to many cancers. It appears crucial for cancer progression, as blood vessels deliver nutrients and oxygen to the neoplastic cells [25]. Furthermore, the microvasculature possibly allows communication between the primary CMM and its metastases. Proangiogenic molecules originate from both cancer cells and the stromal microenvironment. The relative contribution of both compartments is likely to be influenced by the CMM type and site and is balanced by other factors as well [25, 26].

## 7. Conclusion

The multiple interactions between CMM and its stroma are beyond doubt, in particular during the invasive and metastatic phases of evolution. Stromal cells are recognized to secrete metalloproteinases and their inhibitors, growth factors, and other soluble mediators as well participating in the growth and mobility of the CMM cells. In addition, some other molecules are synthesized and overexpressed by stromal cells and/or CMM cells. Immunohistochemistry conveniently identifies Factor XIII-a, *α*1 and *α*5 (IV) collagen chains, versican, elafin, and lysozyme. They possibly influence the migration and thigmotropism of CMM cells including their stem cells.

While CMM cell motility cannot be directly assessed, there is circumstantial evidence that mobility is operative in CMM progression and possibly indicative for prognostic purposes. In addition to the secretion and activation of enzymes modeling the ECM, a variety of stromal changes occur following overexpression of diverse ECM components. Molecular morphology yields evidence suggesting that the CMM stroma plays an integral role in the neoplastic evolution. Although much remains to be learned, the presently summarized changes have diagnostic and prognostic significance that merit to be scrutinized in future works.
